# Remimazolam Anesthesia for a Pediatric Patient With Glutaric Aciduria Type I: A Case Report

**DOI:** 10.7759/cureus.66612

**Published:** 2024-08-10

**Authors:** Tomoko Tsuruno, Hiroki Tateiwa, Yuki Hashimoto, Yoshifumi Katsumata, Takashi Kawano

**Affiliations:** 1 Department of Anesthesiology and Intensive Care Medicine, Kochi Medical School, Kochi, JPN

**Keywords:** mitochondrial disorder, perioperative management, general anesthesia, remimazolam, glutaric aciduria type 1

## Abstract

Glutaric aciduria type I (GA-1) is a rare metabolic disorder caused by an autosomal, recessive, inherited deficiency of glutaryl-CoA dehydrogenase. Reports on the anesthetic management of patients with GA-1 are limited. It has been suggested that inhalation anesthesia is safer than propofol due to the mitochondrial dysfunction inherent in GA-1. However, inhalation anesthesia poses a risk, albeit rare, of malignant hyperthermia, which can result in severe neurological damage in GA-1 patients. Therefore, we considered that management using remimazolam might be effective and, provided a successful general anesthesia using it for a pediatric patient with GA-1. We report a case of a four-year-old girl with GA-1 who underwent a laparoscopic gastrostomy under general anesthesia. Remimazolam was used for both induction and maintenance of anesthesia. Our perioperative management also included measures to prevent a hypercatabolic condition such as adequate hydration and blood glucose control. The patient had an uneventful perioperative course and was discharged on postoperative day 7. Thus, remimazolam is proposed as a new option for anesthetic management in patients with GA-1. Additionally, tailored perioperative management that addresses the unique characteristics of GA-1 is crucial for favorable outcomes.

## Introduction

Glutaric aciduria type I (GA-1) is a mitochondrial metabolism disorder caused by the inherited deficiency of glutaryl CoA dehydrogenase (GCDH) [1]. This enzyme deficiency leads to the accumulation of organic acids, such as glutaric acid and 3-hydroxyglutaric acid, in the brain, which can cause severe neurological damage, particularly during periods of stress or illness [2]. GA-1 typically presents in early childhood, with symptoms including macrocephaly, developmental delay, and dystonia. Acute metabolic crises, often triggered by infections or other stressors, can result in severe and irreversible brain damage [1,2]. Diagnosis is confirmed through urinary organic acid analysis showing elevated levels of glutaric acid and genetic testing for mutations in the GCDH gene. Early diagnosis and management, including dietary restrictions and emergency treatment protocols during illness, are crucial to preventing neurological damage and improving outcomes [3]. Surgery is a risk factor for causing neuronal damage to GA-1 patients, especially for pediatric patients due to the vulnerability of the brain, and careful perioperative management is required [1].

There have been only a few reports on the anesthetic management of GA-1 patients, whereas inhalation anesthetics are considered safer than propofol since GA-1 is classified as a mitochondrial disorder and concerned with a risk of propofol infusion syndrome (PRIS) [4-6]. Historically, there has been concern about the association between mitochondrial disorders and an increased risk of malignant hyperthermia (MH), leading to the avoidance of inhalation anesthesia. Recent evidence, however, indicates that while the risk of MH in patients with mitochondrial disorders might not be as high as previously feared, it is still a consideration [7]. In this regard, remimazolam, a novel benzodiazepine is considered safer, as it poses neither risk. In addition, remimazolam is antagonized by flumazenil, enabling neurological assessment immediately without effects on neurological recovery [8]. Furthermore, remimazolam has been noted to cause less postoperative nausea and vomiting (PONV) than inhalation anesthetics [9,10], a preferable benefit for GA-1 patients. Thus, we chose remimazolam for general anesthesia in a pediatric patient with GA-1. Here, we report a case where we provided attentive perioperative management, including general anesthesia with remimazolam.

## Case presentation

**Figure 1:**
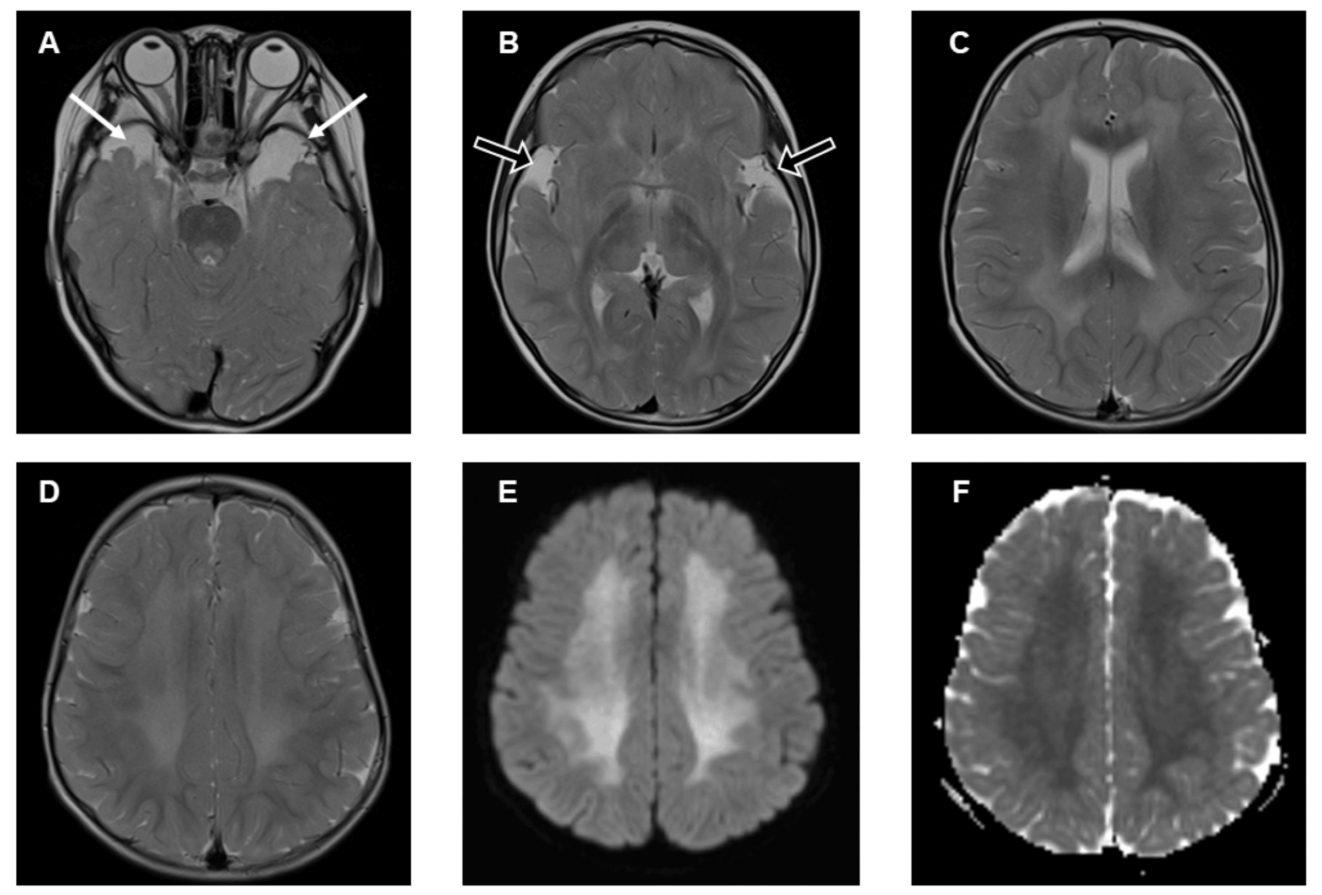
MRI features of the patient at four years old (before surgery) Axial T2-weighted MR images (A-D), a diffusion-weighted image (E), and an apparent diffusion coefficient (ADC) map (F) are shown. Enlarged anterior temporal fossa subarachnoid spaces (white arrows in A) and dilation of the Sylvian fissures (outlined arrows in B) were observed. Diffuse high-intensity signals in T2-weighted images (C, D), corresponding diffusion restriction on the diffusion-weighted image (E), and a drop in the ADC map (F) were observed in the bilateral frontal and temporal white matter. These findings showed almost no change from the MR images taken at birth.

A 4-year-old girl (weight 22 kg, height 109 cm) with GA-1 was scheduled to undergo a laparoscopic gastrostomy under general anesthesia. The patient was suspected of GA-1 with newborn screening, which was subsequently diagnosed following genetic testing. MRI showed the atrophy of the frontotemporal regions of cerebral hemispheres, with enlarged anterior temporal fossa subarachnoid spaces and dilation of the Sylvian fissures, which are common findings for GA-1 patients (Figure 1). 

Additionally, diffuse high-intensity signals in T2-weighted images, with diffusion restriction on diffusion-weighted imaging (DWI), and low apparent diffusion coefficient (ADC) were observed in the bilateral frontal and temporal white matter. No significant changes were noted in the basal ganglia. Her mental development was slightly delayed but physical development was unremarkable owing to the early therapeutic intervention, which included a low-protein diet (lysine and tryptophan eliminated milk) and supplementation with carnitine. As the patient grew, a normal diet was gradually introduced with the concomitant administration of therapeutic milk. Although the therapeutic milk was essential for her treatment, she was unable to ingest it orally due to its taste, and it had to be administered via a nasogastric tube. Each time the tube was accidentally removed, it had to be reinserted, which was a burden for both the patient and her family. Therefore, the decision was made to place a gastrostomy tube.

On the day before surgery, an i.v. line was inserted and a hypotonic solution containing 7.5% glucose was administered to prevent dehydration and hypoglycemia. Additionally, a protein-restricted milk diet was provided until six hours before surgery, and orally ingested glucose solution was allowed until two hours before surgery. The preoperative blood glucose level was 112 mg/dl. No premedication was administered.

**Figure 2:**
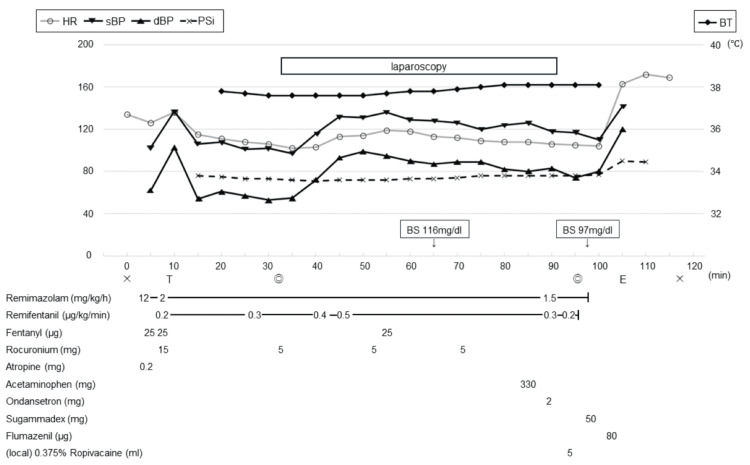
The anesthesia record Cross marks: the beginning and end of anesthesia, T: intubation, E: extubation, Double circles: the beginning and end of surgery. sBP: systolic blood pressure, dBP: diastolic blood pressure, HR: heart rate, PSi: patient state index (their scales are on the left side), BT: body temperature (its scale is on the right side), BS: blood sugar

For the induction of anesthesia, atropine 0.01 mg/kg was administered, and a continuous infusion of remimazolam was started at 12 mg/kg/h. After the loss of consciousness, the remimazolam dose was reduced to 2 mg/kg/h. Subsequently, fentanyl 2 mcg/kg and rocuronium 0.6 mg/kg were administered. Endotracheal intubation was performed without complications. Anesthesia was maintained with a continuous infusion of remimazolam at 1.5-2 mg/kg/h and remifentanil at 0.2-0.5 mcg/kg/min. The depth of anesthesia was monitored using the Sedline® system (Masimo Corporation; Irvine, CA, USA). The patient state index (PSi) remained stable at 60-80. Although this was slightly higher than the usual anesthetic target, hemodynamic parameters were stable. Additionally, boluses of remimazolam and fentanyl were administered, but neither the hemodynamic parameters nor the PSi showed any significant changes. The neuromuscular block was monitored with train-of-four (TOF) stimuli, and rocuronium was added to maintain the TOF count at 0-1. The core temperature was 37.6°C after induction. In patients with GA-1, hyperthermia should be avoided, but shivering due to overcooling is also undesirable. Therefore, we tried to keep it and it was maintained at 37.6-38.0 °C by a warming mattress. The operation time was 60 minutes. During the procedure, an isotonic solution with 5% glucose was provided, and the blood glucose was checked hourly. The target glucose range was set at 80-180 mg/dl and was maintained successfully. Before the end of the surgery, 0.375% ropivacaine was injected into the incision sites as local anesthesia. Additionally, acetaminophen 15 mg/kg and ondansetron 0.1 mg/kg were administered intravenously. Based on the TOF monitor results, sugammadex 2 mg/kg was administered to reverse the neuromuscular block of rocuronium, and the TOF ratio recovered to 100%. After discontinuing the remimazolam infusion, flumazenil 0.004 mg/kg was given, and PSi soared over 90. Simultaneously, spontaneous eye-opening and breathing were confirmed, followed by the removal of the tracheal tube in the operating room. No obvious neuromuscular dysfunction was observed. The anesthesia record is shown in Figure 2.

As postoperative management, acetaminophen 15 mg/kg i.v. every 6 hours and ondansetron 0.1 mg/kg i.v. ever 8 hours for 24 hours postoperatively were provided. The patient had no episodes of unbearable pain, hyperthermia, nausea, or vomiting in the postoperative period. Oral intake and enteral nutrition were initiated as scheduled. The postoperative course was uneventful, and the patient was discharged on postoperative day 7.

## Discussion

GA-1 is a rare inherited disease, and the incidence is estimated to be about 1:90,000-1:120,000 newborns [1]. It is caused by a deficiency of a mitochondrial enzyme, GCDH, which is essential for the degradation of lysine, hydroxylysine, and tryptophan. Dietary management, such as a low-protein diet restricted in lysine/tryptophan, and supplementation with carnitine/riboflavin is the main treatment for GA-1 patients. In addition, emergency treatment during events that promote catabolism, such as fever, vomiting, infection, and surgery, is required to avoid acute encephalopathic crises [1]. Therefore, attentive management-confirmed emergency treatment is required in the perioperative period.

There are a few reports of general anesthesia for GA-1 patients, and most recommend inhalation anesthetics [4,5] since propofol could cause PRIS, especially in patients with mitochondrial disorder [11]. On the other hand, successful anesthetic management with total intravenous anesthesia with propofol for GA-1 patients had been reported [6], and it suggested avoiding inhalation anesthetics due to the potential link between mitochondrial disorders and MH. Recent studies indicate that the direct link between mitochondrial disorders and MH susceptibility is weaker than previously thought [12]. Nevertheless, careful anesthetic management remains crucial to prevent complications [7]. If MH does occur, even unlikely, in GA-1 patients, the metabolic crisis can exacerbate mitochondrial dysfunction, leading to severe and potentially irreversible damage, particularly in the brain.

Considering these problems, remimazolam is an optimal anesthetic as benzodiazepines are generally safe for patients with these conditions [7,12,13]. Remimazolam is a novel benzodiazepine anesthetic that is hydrolyzed by non-specific tissue esterase rapidly and provides short-acting. In addition, it is antagonized by flumazenil, which allows for safer management. Unlike propofol, remimazolam should not cause PRIS. Furthermore, benzodiazepine is considered a safe drug for MH [14]. There are reports of patients at risk for MH being managed under general anesthesia with remimazolam without complications [7,15].

Although there is limited data available on remimazolam for pediatric patients, observational studies and case reports have gradually proven its safety in pediatric anesthesia [7,15-18]. Furthermore, it was demonstrated that the pharmacokinetics of remimazolam with continuous infusion in pediatric patients are similar to those in adults and indicated that remimazolam has favorable characteristics for pediatric anesthesia as well [19]. In this case, flumazenil was used to antagonize remimazolam to assess the neurological function clearly. No neurological complications were confirmed, and the response to command was good. However, flumazenil could antagonize the anticonvulsants of benzodiazepine and should be carefully used if the patients use anticonvulsants. The safety of remimazolam and flumazenil for patients with neurological diseases is propounded whereas further studies are warranted [8].

Preventing hypercatabolism, which can result in an encephalopathic crisis, is crucial in managing GA-1 patients. Conditions accelerating catabolism, such as hypoglycemia, dehydration, hyperthermia, intolerable pain, and vomiting, must be avoided [1,4]. In this case, to avoid hypoglycemia and dehydration, the i.v. line was kept on the day before surgery and adequate fluid and sugar were provided. The patient had been hospitalized several times for emergency treatment for infectious disease and fever, and that protocol was referenced for this perioperative fluid management. Blood glucose levels were monitored and maintained within the target range. Following a preoperative discussion between the anesthesiologists and the patient's primary doctor, the target blood glucose range was set at 80-180 mg/dl in accordance with the guidelines [1]. The body temperature was already slightly high after the induction. If it was cooled too much, it could cause shivering, which triggers a neurologic crisis. Therefore, we decided to control it at the same level. It was achieved and no shivering was observed. In addition, acetaminophen was given every 6 hours for 24 hours postoperatively not only for analgesia but also for the prevention of hyperthermia. Furthermore, PONV should be prevented, and ondansetron was administered before the end of surgery and every 8 h for 24 h postoperatively. Recent reports indicate that remimazolam causes less PONV than inhalation anesthetics in adults [9,10], which is an additional advantage of remimazolam. While specific studies focusing on the reduction of PONV in pediatric patients using remimazolam are limited, general findings suggest that remimazolam has a comparable adverse reaction profile to other benzodiazepines, with common side effects including vomiting [20].​ Owing to these interventions, no neurological complication was observed.

## Conclusions

We successfully performed general anesthesia with a total intravenous infusion of remimazolam for a pediatric patient with GA-1. The characteristics of remimazolam are suitable for general anesthesia in patients with GA-1, and it is considered to be a viable option. Multimodal perioperative management, which includes not only appropriate anesthetic selection but also the prevention of hypercatabolism, is essential for patients with GA-1.
